# Gaming-Based Tele-Exercise Program to Improve Physical Function in Frail Older Adults: Feasibility Randomized Controlled Trial

**DOI:** 10.2196/56810

**Published:** 2024-11-27

**Authors:** Lakshmi Kannan, Upasana Sahu, Savitha Subramaniam, Neha Mehta, Tanjeev Kaur, Susan Hughes, Tanvi Bhatt

**Affiliations:** 1 College of Applied Health Sciences University of Illinois at Chicago Chicago, IL United States; 2 College of Medicine, Geriatrics University of Illinois at Chicago Chicago, IL United States; 3 Center on Health Research on Aging School of Public Health University of Illinois at Chicago Chicago, IL United States

**Keywords:** exergame training, Matter of Balance, MOB, pre-frail, tele-exergame, tele-rehabilitation, gaming-based, tele-exercise, physical function, frailty, older adults, aging, physical activity, dementia, CogXergaming, telehealth, dynamic balance

## Abstract

**Background:**

Frailty leads to reduced physical activity can cause increased fall risk. This contributes to accelerated aging processes, leading to adverse health outcomes and reduced quality of life. We have developed and piloted the design, usability, safety, and feasibility of a gaming-based cognitive-motor (CogXergaming) tele-exercise protocol in prefrail older adults.

**Objective:**

This pilot randomized control trial tested preliminary feasibility and effectiveness of the CogXergaming telehealth protocol for improving physical function.

**Methods:**

Community-dwelling, prefrail older adults were randomly assigned to CogXergaming (n=13) or a control group (n=14). The CogXergaming group received supervised tele-exercises in a gaming format for 6 weeks (3 sessions per week) comprising 18 sessions lasting 90 minutes each. Control group participants participated in a Matter of Balance (MOB), an 8-week, once-a-week structured 90-minute tele-session that has been shown to reduce the fear of falling and increase physical activity. Feasibility of training was obtained by computing the median duration of training sessions for the CogXergaming group. Effectiveness was assessed using dynamic balance control (Four Square Step Test), subjective self-efficacy (Activities-Specific Balance Confidence scale), gait function (Tinetti Performance Oriented Mobility Assessment), muscle strength (30-second chair stand test), and endurance (2-minute step in-place test).

**Results:**

Of the 45 participants enrolled in the study, 4 participants from CogXergaming group and 5 from MOB group lost contact after signing the consent form and did not receive their respective intervention. Eighteen participants were randomized to each group. In the CogXergaming group, 15 (83%) completed the intervention, with 3 (16%) dropping out in the first week. In the MOB group, 16 (88%) completed the program, with 2 (11%) withdrawing during the first week. In addition, there was a significant time group interaction for Four Square Step Test (*F*_1,21_=5.55, *P*=.03), Tinetti Performance Oriented Mobility Assessment (*F*_1,25_=4.16, *P*=.05), and 30-second chair stand test (*F*_1,21_=5.06, *P*=.03), with a significant improvement in these measures for the CogXergaming group post training, compared with no change observed in the MOB group.

**Conclusions:**

These pilot findings indicate that CogXergaming is feasible and applicable in prefrail older adults. Such game-based protocols can be beneficial in improving physical function among community-dwelling, prefrail older adults, however, the efficacy of such training requires further investigation.

**Trial Registration:**

ClinicalTrials.gov NCT04534686; https://clinicaltrials.gov/study/NCT04534686

## Introduction

Frailty is an age-associated biological syndrome with a prevalence of about 4% to 16% of community-dwelling older adults aged 65 years or older in the United States [1-5]. It significantly affects reserves needed for physical, cognitive, psychological, and social function through loss of muscle mass and weakness, increased fatigue and exhaustion, reduced gait speed, unintended weight loss, reduced physical activity, and cognitive impairment. Specifically, frail, and prefrail older adults are respectively at 3- and 1.36-times higher risk of falling compared with healthy older adults. Consequences of these falls like fractures, head injuries, etc, result in higher health care use and costs, and frequent hospital admissions. All these can further debilitate physical functioning; ultimately resulting in long-term disability overall burdening the health care system [6-11]. Although older adults who are prefrail do not experience these consequences, prefrailty presents an opportunity to reverse or avoid the conversion to frailty [1,4,12]. Thus, developing targeted fall prevention programs for prefrail individuals could mitigate frailty progression and alleviate health care burdens.

Conventional fall prevention programs like Matter of Balance (MOB) [13], Otago [14], Stepping on [15], Tai Chi [16], Stay Active Independent for Life [17], Young Men’s Christian Association moving for better balance [18], FallScape [19], Capable [20], and FallTalk [21] have demonstrated promising effects on reducing fall risk among older adults. These programs have been virtually translated to overcome geospatial barriers, making them more accessible to the community [22]. In addition, they are specifically designed to educate older adults about falls and share methods to prevent falls using exercises specific to balance control [23], muscle strength [24], flexibility [25] which, together, can reduce fear of falling [26], and increased balance confidence [27]. For example, the MOB program has demonstrated adaptability and long-term implementation effects while being cost-effective with robust beneficial effects among ambulatory older adults. Studies have reported significant improvement in balance control [26]; gait speed [28]; and reduced fear of falling [29,30], number of falls [13], number of fallers [31], and hospital admissions. However, the program requires a certified trainer to administer it, which limits its use in daily practice. Second, current fall risk reduction programs do not address cognitive function, which is often associated with reduced balance control and higher fall risk among older adults [32-34]. Therefore, there is a need to design and test fall prevention programs for prefrail older adults that are more accessible and cover all aspects of fall risk, targeting both physical and cognitive functions.

Alternate forms of therapy like exergaming (ie, the use of games for exercise) interventions have demonstrated beneficial effects mitigating fall risk factors, as well as improving physical and cognitive function [35-37]. Exergames provides immediate biofeedback (ie, visual, sensory, and auditory) that keeps participants engaged and motivated and thus, improves therapy adherence. Commercial devices like Microsoft Kinect, Nintendo (Wii), Sony PlayStation Move, etc, are often used to access exercise regimens in that use of dance, sport-like activities, balance board activities, and upper extremity movements without the need to leave home [38,39]. However, due to the rapid advancements of commercial gaming devices, they may not be accessible, and are not user friendly for older adult populations, and target only younger adults. As a result, older adults have increasingly relied on web- or tele-based fitness applications (ie, YouTube [Google] videos, physical activity applications) for maintaining physical activity levels while at home [40,41]. However, web-based, and tele-health programs vary significantly with respect to the type, intensity, duration, and frequency of exercise followed resulting in heterogeneity of interventions. Finally, there are no standardized assessment methods to determine adherence and feasibility to web-based programs. Our previous work administered a tele-based assessment and training method called CogXergaming, that is safe to deliver to older adults [42]. The training involves remotely monitoring older adults who mimic a combination of exergame videos (noninteractive) that targets training of balance control, endurance, cognitive-motor function, and mobility [42]. The observed benefits suggest initial safety and feasibility implications in older adults. Adopting a prudent, stepwise approach to assessing the feasibility of a similar training paradigm in prefrail older adults is essential for minimizing potential harm to frail individuals. This approach will ensure rigorous evaluation to establish the safety and effectiveness of interventions before broader implementation in higher-risk populations.

Therefore, this pilot used a NIH (National Institutes of Health) stage 1 model, randomized control trial to investigate the feasibility and effectiveness of a noninteractive, virtually delivered (through Zoom; Zoom Video Communications) exergame (ie, CogXergaming) compared with virtually delivered (also through Zoom) MOB on physical function (ie, balance control assessed through Four Square Step Test (FSST), self-efficacy through Activities-Specific Balance Confidence (ABC) scale, gait function through Tinetti Performance Oriented Mobility Assessment (POMA), muscle strength through 30-second chair stand test (30-CST), and endurance through 2-minute step test) among prefrail older adults. We hypothesized that this pilot study will be feasible and that participants will demonstrate adherence to the CogXergaming program. In addition, the study will explore between-group (CogXergaming vs MOB) differences to gather preliminary data on the comparative effectiveness of the 2 interventions.

## Methods

### Participants

This NIH stage 1, pilot study involved 50 community-dwelling older adults older than 60 years interested in the study with 45 older adult enrolled after virtually obtaining a written informed consent (through Docusign). The study was registered on ClinicalTrials.gov (#NCT04534686). Participants were recruited from rehabilitation clinics and senior housing sites in the Chicago metropolitan area.

### Ethical Considerations

The institutional review board (IRB) of the University of Illinois at Chicago approved the study (#2020-0280).

### Participant Eligibility

Prefrail older adults were recruited. They were characterized as prefrail if at least 2 out of the 3 criteria were present: (1) self-reported unintentional weight loss (>10 lbs) in the last 1 year [1], (2) self-reported general fatigue >3 on rate of perceived exertion (ie, according to Borg’s scale ranging from 1-10 with 10 being the maximum perceived exertion) at rest [1], and (3) reduced physical activity levels (ie, exercise less than 3 hours a week) [1]. These criteria are recognized as preliminary indicators of prefrailty [43,44] and were selected as feasible measures for remote screening, aligning with the study’s practical considerations and the goal of targeting prefrail individuals for our intervention. Participants were required to possess a laptop or tablet or personal computer with stable internet provision. This decision was made to focus on the need for larger screens that could effectively support the tasks and activities involved in our study. In addition, participants must be able to walk more than 30 feet, stand without an assistive device for at least 5 minutes (length of an exergame was approximately 1-3 minutes), and understand English. Participants were excluded if they self-reported any acute and uncontrolled chronic medical illnesses such as cardiovascular or pulmonary conditions; were recently hospitalized (<3 months); or had a diagnosed neurological condition such as a stroke or Parkinson disease. They were also excluded if their weight exceeded 100 kg and if they were using sedatives or undergoing psychiatric treatment that could affect their cognitive and physical performance during training sessions.

Out of 50 OA who met the inclusion criteria, 45 were included in the study and signed the consent form. Participants were randomized to either of the training groups (n=22 in CogXergaming and n=23 in MOB) by flipping a coin by a research coordinator. This is a simple and transparent randomization technique based on the outcome of a coin toss. A specific research assistant was assigned to administer the CogXergaming intervention, while a different research assistant, trained in delivering the MOB training, administered the MOB intervention. These research assistants did not interact with participants assigned to the other intervention to prevent any potential bias in intervention delivery. However, we also had attrition (n=9) between consenting and start of intervention due to losing contact with participants (unreachable, phone number changed, changed mind, and not interested).

### Research Design

#### Tele-Based Training: CogXergaming

Safety was a priority; therefore, the researcher inspected the environment through the Zoom call and asked the participant to clear away any objects that could potentially cause injury. The researcher instructed the participant to choose a room with good lighting. Besides the break that automatically occurred between subsessions, additional rest breaks were provided upon participant requested ones.

Participants were trained one-on-one for 90 minutes per day and 3 times per week for 6 weeks (for a total of 18 sessions). The intervention was comprised of 4 subsessions that sequentially targeted balance control, endurance, cognitive-motor function, and mobility. Balance control involved mimicking Wii-fit games such as “Super Hula Hoop,” “Boxing,” “Basic Step,” and “Soccer Head.” Endurance involved mimicking Just Dance videos: “Price Tag,” “Party Rock Anthem,” “I Was Made for Loving You,” and “Dynamite.” Cognitive-motor function involved responding to customized games: “Arithmetic Stepping,” “Finding the Bar,” “Stepping Colors,” and “Paired Association Task.” The mobility domain involved mimicking certain yoga and tai-chi movements. Participants were instructed to mimic each video shown to them while maintaining the pace. The first and last 10 mins of each session were comprised of warm-up and cool-down exercises. The researcher shared their screen, and participants were required to have their video turned on to enable the researcher to observe their performance.

The length of the sessions remained on an average 85-90 minutes. Over the training weeks, participants needed lesser rest breaks and were able to progress from light to moderate to more intensive games (challenging balance control, ie, progression from standing dynamic weight shifting to stepping in place to diagonal stepping to side shuffling). More details of this method have been previously published and have shown feasibility [42].

#### Matter of Balance

All sessions were conducted on Zoom in compliance with HIPAA (Health Insurance Portability and Accountability Act). Participants were trained in groups of 10. Each session lasted 120 minutes and was performed 1 time per week for 8 weeks. The researcher administering MOB was certified to perform MOB training. Each MOB session included a discussion of practical coping strategies, group problem-solving, home safety evaluation strength exercises of the lower limb, coordination exercises, and standing balance exercises [26].

### Tele-Assessment

All tele-based assessment tests have shown to be feasible and were described in our previous study [42]. For the current study, we used time taken to complete the FSST with lower numbers (in seconds), indicating better performance to assess balance control [45]. Subjective self-efficacy questionnaire was assessed by the ABC scale with higher values indicating higher confidence levels [46]. Gait function was assessed using the Tinetti POMA with higher scores indicating better performance [47,48]. To assess muscle strength, we used the number sit-to-stands on the 30-CST with higher values indicating greater lower-limb muscle strength [49]. Endurance was assessed using the total number of steps completed on the 2-minute step test with higher numbers indicating better endurance [50].

### Statistical Analysis

All analysis was performed using SPSS (version 24; IBM Corp). To assess demographic differences, an independent *t* test with Bonferroni correction for all characteristics except gender. A chi-square test of independence for gender was conducted. It should be noted that this pilot trial was designed to primarily assess the feasibility and safety of a tele-exercise intervention (CogXergaming) in prefrail older adults. The study was not powered for formal efficacy analyses due to the limited sample size and exploratory nature of the investigation. A 2×2 repeated-measure ANOVA was performed to determine the effect of time (before and after training), group (CogXergaming vs MOB), and the time group interaction on all tele-assessments (ie, balance control and confidence, gait function, muscle strength, and endurance). Finally, median duration of training sessions for the CogXergaming group was computed to address feasibility of training. Significant main effects were resolved by post hoc tests with Bonferroni correction with significance level =.05.

## Results

### Demographics

Participant descriptive characteristics are shown in Table 1. No significant group differences in participant characteristics were observed (*P*>.05).

**Table 1:** Participant demographic characteristics for both groups.

Demographics	CogX^a^ group (n=13; 6 male and 7 female), mean (SD)	MOB^b^ group (n=14; 4 male and 10 female), mean (SD)	*P* value
Age (years)	72.07 (4.75)	71.14 (4.76)	0.61
Weight (kg)	72.64 (15.95)	70.25 (13.78)	0.94
Height (m)	1.61 (0.10)	1.68 (0.06)	0.65
BMI	27.83 (6.05)	25.07 (5.6)	0.29
Years of education	16.15 (2.91)	15.5 (2.53)	0.58

^a^CogX: CogXergaming.

^b^MOB: Matter of Balance.

### Use of Tele-Based Assessment and Training

Participants possessed either a tablet or laptop to participate in the intervention activities. However, the specific data on the distribution between tablet or laptop usage among participants were not obtained. While acknowledging the importance of reporting device distribution for comprehensive study outcomes, our focus was primarily on the suitability of tablet or laptop devices to achieve the study objectives effectively.

**Figure 1:**
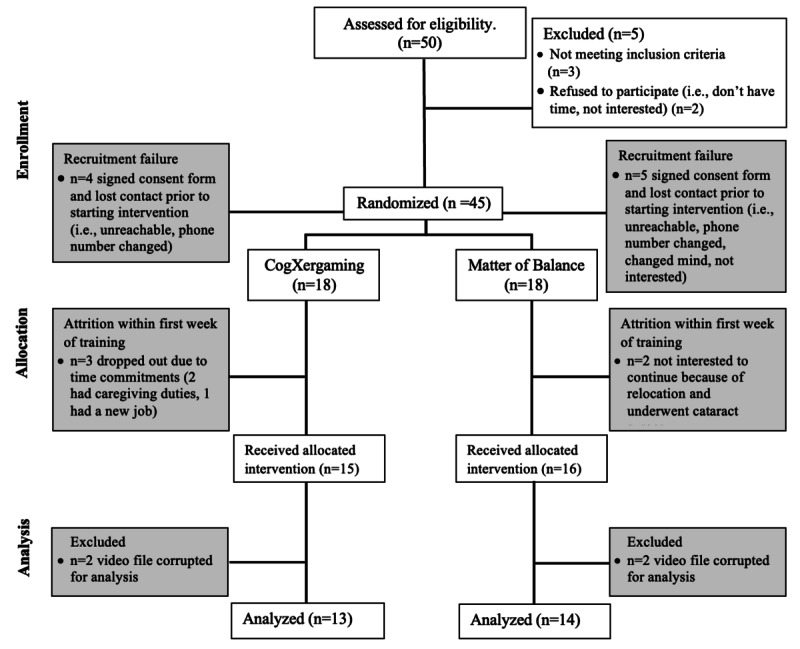
Research design.

Of the 45 participants enrolled in the study, 4 participants from CogXergaming group and 5 participants from MOB group lost contact after signing the consent form and did not receive their respective intervention (Figure 1). This primarily was a recruitment failure. Both the tele-based assessment and training were performed through the HIPAA-compliant Zoom application. An individualized Zoom link was sent to each CogXergaming participant for their assessments and training, and a common Zoom link was sent individually for MOB group sessions to protect participants’ personal information. All participants were provided with detailed instructions over the phone, guiding them through the process of connecting to the Zoom platform. This approach facilitated seamless participation in virtual sessions, with no notable issues encountered during setup. All assessment and the training sessions were recorded for postsession analyses.

### Feasibility

Of the 18 people who were randomized to the CogXergaming group, 15 (83%) successfully completed the intervention. All the attrition from participation in the treatments tested occurred within the first week of both programs. During the first week of CogXergaming, 3 (16%) participants withdrew or dropped out due to lack of time and commitment (eg, got a new job, caregiving duties). Similarly, of the 18 people randomized to MOB, 16 (88%) completed the program. Specifically, 2 people (11%) were not interested in continuing after attending the first session of the MOB group. We did not experience attrition from treatment in either group after participants completed the first week of training.

### Effect of Training on Dynamic Balance

**Figure 2:**
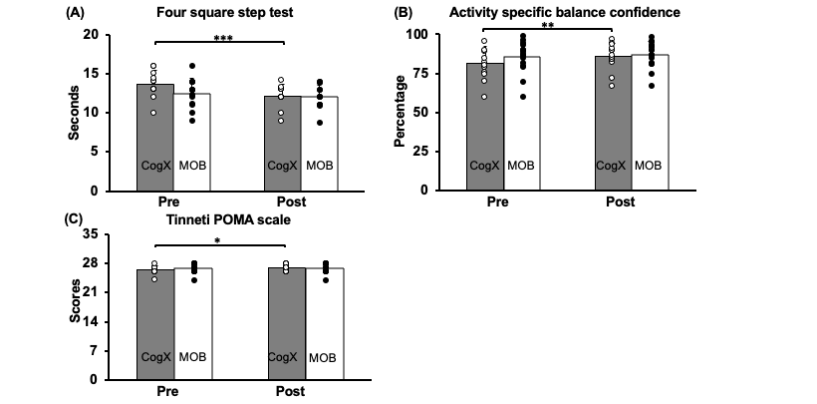
Effect of CogXergaming and Matter Of Balance training in prefrail older adults on balance control and gait function. Figures represent mean (SDs) of pre- to posttraining changes on (A) four square step test, (B) activities-specific balance confidence scale, and (C) Tinetti performance-oriented mobility assessment. Significantly greater performance is indicated by *P<.05, **P<.01, ***P<.001.

For the FSST, the 2×2 repeated measures ANOVA showed a significant main effect of time (*F*_1,21_=19.14, *P*<.001) and group time interaction (*F*_1,21_=5.55, *P*=.03) on the time taken to complete the test. However, no group effect was observed (*F*_1,21_=0.33, *P*=.57). Resolving for the significant main effect of time and the interaction, paired within group *t* tests revealed a significant reduction in time taken to complete the FSST in the CogXergaming group (*t*_10_=4.84, *P*<.001) post training, however, no significant reduction of time was observed in the MOB group (*t*_11_=1.41, *P*=.18; Figure 2A).

### Effect of Training on Subjective Self-Efficacy

For the ABC, the 2×2 repeated measures ANOVA showed a significant main effect of time (*F*_1,25_=12.03, *P*<.01) on the balance confidence score. However, no significant group time interaction (*F*_1,25_=3.05, *P*=.09) or group effect (*F*_1,25_=0.65, *P*=.42) was observed. Resolving for main effect of time, a paired *t* test showed a significant improvement in balance confidence score within the CogXergaming group (*t*_12_=–2.92, *P*=.01), but no improvement within the MOB group (*t*_13_=–1.75, *P*=.10) was observed (Figure 2B).

### Effect of Training on Gait Function

The 2×2 repeated measures ANOVA showed a significant main effect of time (*F*_1,25_=7.77, *P*=.01) and significant group time interaction (*F*_1,25_=4.16, *P*=.05) on the POMA score. However, we did not see a group effect (*F*_1,25_=0.46, *P*=.5). Resolving for main effect of time and interaction, the paired *t* test showed a significant improvement in the POMA score within the CogXergaming group (*t*_12_=–2.52, *P*=.03) but no within group improvement in the MOB group (*t*_13_=–1.0, *P*=.33) was observed (Figure 2C).

### Effect of Training on Lower Limb Muscle Strength

**Figure 3:**
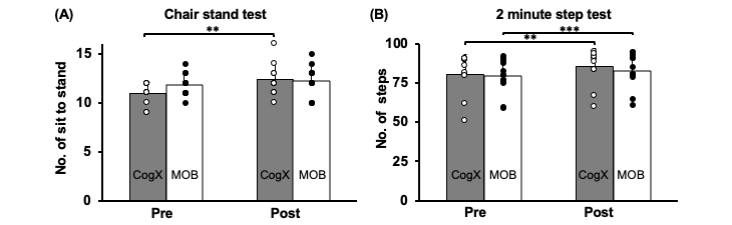
Effect of CogXergaming and Matter Of Balance training in prefrail older adults on lower limb muscle strength and endurance. Figures presented are mean (SDs) of pre- to posttraining scores on (A) 30-second chair stand test (30-CST) and (B) 2-minute step test for both groups. Significantly greater performance is indicated by *P<.05, **P<.01, ***P<.001.

The 2×2 repeated measures ANOVA showed a significant main effect of time (*F*_1,21_=16.45, *P*<.001) and significant group time interaction (*F*_1,21_=5.06, *P*=.03) indicating that both groups performed an increased number of sit-stands on the 30-CST. However, there was no significant main effect of group (*F*_1,21_=0.10, *P*=.75). Resolving for main effects and interaction, the paired *t* test showed a significant increase in the number of sit-to-stands on the 30-CST within the CogXergaming group (*t*_10_=–3.73, *P*<.01); however, there was no significant within group improvement observed in the MOB group (*t*_11_=–1.6, *P*=.13; Figure 3A).

### Effect of Training on Endurance

The 2×2 repeated measures ANOVA of the 2-minute Step Test scores showed a significant main of effect of time (*F*_1,21_=37.22, *P*<.001) with no significant group time interaction (*F*_1,21_=2.57, *P*=.12) or group effect (*F*_1,21_=0.26, *P*=.61) on number of steps completed. Resolving for main effect of time, paired *t* tests revealed a significant improvement in number of steps on this measure within the CogXergaming group (*t*_10_=–3.97, *P*<.01), and the MOB group (*t*_11_=–6.23, *P*<.001, Figure 3B).

## Discussion

### Principal Findings

This pilot randomized controlled trial investigated the feasibility and effectiveness of a tele-based, noninteractive CogXergaming training compared with tele-based MOB program on physical function in prefrail older adults. The results showed that the intervention was feasible as shown by tolerability of the intervention with a lack of any expected and unexpected intervention-induced adverse events. The intervention also showed promise of being effective as seen by the significantly and differentially greater improvements in physical function within the CogXergaming group. Specifically, we observed significant and differential within group improvements within the CogXergaming group in balance control and confidence, gait function, and lower limb muscle strength. While both groups showed significant improvement in endurance, the benefits were greater among the MOB group. These results suggest that the tele-based, noninteractive CogXergaming intervention has the potential to improve physical function among prefrail older adults.

### Feasibility

The significant and differential improvements observed in the CogXergaming group in 3 of the 4 outcomes assessed underscore the potential of mimicking video-based interventions to enhance physical function among prefrail older adults. The time demand posed a notable challenge in our study. Notably, participant dropout rates observed largely attributed to personal commitments among both groups. Specifically, the CogXergaming intervention necessitated a higher frequency, occurring thrice weekly over a span of 6 weeks, in contrast to the MOB program, which occurred once weekly over 8 weeks. While the MOB intervention underscores the importance of exercise adherence among prefrail older adults, it relies on the presence of a health coach. Conversely, the CogXergaming intervention offers participants the flexibility to engage at their convenience, potentially mitigating scheduling conflicts. However, the implications of unsupervised engagement on compliance remain uncertain and may require further investigation. In addition, the physiological underpinning of this approach remains to be explored to understand the mechanisms of actions that confer benefits.

The CogXergaming group had high adherence, similar to the MOB group, demonstrating its feasibility as a remote intervention. Additionally, both groups experienced similar levels of attrition, which occurred only during the first week of training. This early dropout may be due to the prefrail characteristics of the participants. CogXergaming, with its gamified approach, may offer additional engagement to participants and flexibility to clinicians for tailorizing the intervention. The challenge of moving from a sedentary lifestyle, compounded by psychological factors such as fatigue and depression, may impede even highly motivational exercise programs. The intensity and dosage of exercises in the CogXergaming group, if increased, could potentially help maximize the benefits. An enhancement of the program could address essential components of physical function, including balance control, muscle strength, endurance, and cognitive function. In addition, few technical challenges were encountered, as all prefrail older adults successfully connected to the Zoom link. This success may be attributed to the step-by-step guidance provided during phone calls. These findings support the implementation of this approach in future studies and clinical applications. However, implementing a similar training approach on a larger scale could enhance our ability to interpret qualitative responses more effectively. With emerging exergaming devices commercially, the development of innovative strategies that surmount geospatial barriers is crucial in facilitating physical function in prefrail older adults. The availability of these approaches could promote home-based, cost-effective, user-friendly methods of intervention delivery that improve accessibility and adaptability in the community.

### Effect of Training on Dynamic Balance, Subjective Self-Efficacy, and Gait Function

A time effect and a group time interaction was observed in the FSST, ABC, and POMA scale with the CogXergaming group displaying greater improvement in balance control post training compared with the MOB group. Specifically, FSST and POMA measures the temporal characteristics of adjusting postural stability during forward, backward, and side stepping. This task requires lower limb strength, ankle stability, hip and trunk control, core strength (in the abdominal and lower back muscles), and sensory system function (ie, proprioception, visual, and vestibular). The series of subsessions involved during a CogXergaming session promoted weight shifts, stepping, trunk rotations, and short durations of standing on 1 leg within a fixed time limit. These activities were holistic while being task-specific, optimizing motor and interlimb coordination with specific movements, promoting quick multidimensional weight shifts. Consistent practice of these movements over 18 sessions may have been enough to promote motor learning and skill retention that could have attributed to the reduced time required to complete FSST and POMA. In addition, in our study, the participants engaged in CogXergaming within a familiar environment, that is, their own home. This environmental familiarity with each game to be played within a fixed time limit could have contributed to increased comfort levels during training, enabling participants to focus more on mastering specific motor movements such as forward, backward, and side stepping. Previous studies have explored the concept of perceptual-motor recalibration in familiar environments [51,52]. These studies report that when individuals are in a familiar environment, the sensory demands from visual and vestibular systems, relying more on proprioception input and thus, prioritizing sensory resources toward maintaining postural stability, are reduced. However, there was no prominent reduction of spatiotemporal gait characteristics among the MOB group. The MOB fall prevention education program was also provided to participants in their homes. However, MOB participants may not have practiced exercises consistently as they were advised to leading them to experience no change in physical activity levels. In addition, it is noteworthy that despite the insignificant differences between the groups at baseline, the MOB group displayed potential ceiling effects for the FSST, ABC, and POMA. This suggests that participants in the MOB group may have started the intervention at a higher baseline level of function, potentially limiting observable improvements on the scales used. However, given our sample size limitations, definitive conclusions regarding a ceiling effect cannot be drawn at this stage.

### Effect of Training on Lower Limb Muscle Strength

There was a main effect of time and an interaction effect between time by group such that the CogXergaming group showed a significant increase in the number of sit-to stands on the 30-CST, demonstrating greater muscle strength in lower limbs, compared with no change on this outcome within the MOB group. The CogXergaming training involved mimicking complex motor movements shown in videos of tai chi, yoga, and dance [42]. These movements predominantly involved moving the lower limb in multiple directions and maintaining postural stability while moving the upper extremity. This approach could have reinforced the ability of prefrail older adults to consistently participate on the lower extremity during training for 18 sessions. Since prefrail older adults exhibit muscle weakness, these complex activities provided a medium to gradually improve muscle strength, which helped CogXergaming participants to perform better post training. On the other hand, since MOB group notably focused on psychosocial aspects of educating older adults about prevention of falls, its ability to improve muscle strength may have been limited. MOB was designed to promote a holistic understanding of fall prevention by encouraging participants to consider physical capabilities, balance control, environmental awareness, and the importance of physical activity. Furthermore, the higher number of sit-to-stands observed in the MOB group could suggest a potential ceiling effect among participants, indicating that they may have started the intervention at a higher level of functioning compared with participants in the CogXergaming group. However, the long-term effects of MOB on muscle strength need to be explored further in prefrail older adults.

### Effect of Training on Endurance

Despite no time group interaction effect, there was a time effect with both groups that displayed significant improvement in endurance on the 2-minute step test. The CogXergaming intervention was designed to mimic exergames focused on several stepping and motor movements performed at a medium-paced intensity that could have attributed to the improvement on the 2-minute step test. The incorporation of dance movements, cognitive-motor function training, and balance control exercises during the exergaming training sessions also targeted cognitive demands on motor performance. This feature could have inadvertently increased the overall pace of exercises performed across the sessions, particularly as participants tried to complete various movements within stipulated time limits, placing an added emphasis on quick movement completion that potentially elevated heart rates. The consistent performance of activities under such conditions over 18 sessions likely facilitated participants’ adaptation to performing exercises at a specific intensity. Notably, the posttraining improvement observed in the number of steps completed within 2 minutes aligns with the practiced activity of quickly completing the task to mimic the training. These results indicate that it is possible to improve aerobic capacity merely by mimicking and matching movements at the intensity demonstrated in the videos for prefrail older adults. Furthermore, the selection of music with a specific pace for the dance component in the CogXergaming group could have induced significant improvements in endurance. The finding that the endurance gains in the MOB group were greater than those seen in the CogXergaming group in the same test performance, through education alone, is noteworthy. While participants were not monitored for their physical activity levels post training, this finding indicates that more studies are needed to investigate the lasting impact of education on physical activity levels in this population.

### Limitations

Apart from the benefits observed, acknowledging the limitations of the study is essential. First, the relatively low sample size in our pilot study meant that the study did not have the statistical power to enable valid between group efficacy testing. The differential within-group improvements on 3 of the outcomes is promising and warrants follow-up in a fully powered future. The nonequivalence between the groups in our study may have influenced the outcomes observed. However, since this was a pilot study aimed at evaluating the feasibility of a noninteractive exergaming training, future research efforts may benefit from designing equivalent-based training programs to determine the efficacy of the intervention more accurately. Second, caution must be used in generalizing the observed results to other populations. Higher sample size in future studies may provide more robust and representative data, particularly in terms of the scope and methods used to identify prefrail individuals. Third, the intervention implicitly challenged cognitive function but did not include comprehensive examination of cognitive aspects associated with increased risk of falling (such as reaction time, working memory, and executive function). Further exploration of these additional outcomes is therefore warranted. Finally, although the study included prefrail older adults, we did not differentiate between physical frailty, cognitive frailty, or both. Future investigations may benefit from classifying participants based on these distinct frailty profiles to assess intervention effects more powerfully. In conclusion, while our NIH stage 1 pilot study provides valuable insights into the feasibility of a noninteractive exergaming intervention, future research efforts should address these limitations to advance our understanding of the intervention’s efficacy and generalizability.

### Conclusion

This pilot randomized control trial provides preliminary findings that the 6-week CogXergaming intervention has the potential to improve physical function among prefrail older adults. Specifically, the results show differential within group significant improvements in balance control, self-reported balance confidence, and muscle strength after participation in CogXergaming. Effects were also seen in endurance, but they were not as large. These findings indicate that CogXergaming could potentially help reduce the risk of falls while promoting physical activity among prefrail older adults. Future adequately powered studies of this promising program are needed to demonstrate efficacy among prefrail older adults.

## Appendix

Multimedia Appendix 1 CONSORT-eHEALTH checklist (V 1.6.2).

## Abbreviations

30-CST: 30-second chair stand test
ABC: Activities-Specific Balance Confidence
FSST: Four Square Step Test
HIPAA: Health Insurance Portability and Accountability Act
IRB: institutional review board
MOB: Matter of Balance
NIH: National Institutes of Health
POMA: Performance Oriented Mobility Assessment

## References

[1] Fried LP, Tangen CM, Walston J, Newman AB, Hirsch C, Gottdiener J, Seeman T, Tracy R, Kop WJ, Burke G, McBurnie MA, Cardiovascular Health Study Collaborative Research Group (2001). Frailty in older adults: evidence for a phenotype. J Gerontol A Biol Sci Med Sci.

[2] Kiely DK, Cupples LA, Lipsitz LA (2009). Validation and comparison of two frailty indexes: The MOBILIZE Boston Study. J Am Geriatr Soc.

[3] Bandeen-Roche K, Xue QL, Ferrucci L, Walston J, Guralnik JM, Chaves P, Zeger SL, Fried LP (2006). Phenotype of frailty: characterization in the women's health and aging studies. J Gerontol A Biol Sci Med Sci.

[4] Woods NF, LaCroix AZ, Gray SL, Aragaki A, Cochrane BB, Brunner RL, Masaki K, Murray A, Newman AB, Women's Health Initiative (2005). Frailty: emergence and consequences in women aged 65 and older in the Women's Health Initiative Observational Study. J Am Geriatr Soc.

[5] Cawthon PM, Marshall LM, Michael Y, Dam TT, Ensrud KE, Barrett-Connor E, Orwoll ES, Osteoporotic Fractures in Men Research Group (2007). Frailty in older men: prevalence, progression, and relationship with mortality. J Am Geriatr Soc.

[6] Trombetti A, Reid KF, Hars M, Herrmann FR, Pasha E, Phillips EM, Fielding RA (2016). Age-associated declines in muscle mass, strength, power, and physical performance: impact on fear of falling and quality of life. Osteoporos Int.

[7] Deary IJ, Corley J, Gow AJ, Harris SE, Houlihan LM, Marioni RE, Penke L, Rafnsson SB, Starr JM (2009). Age-associated cognitive decline. Br Med Bull.

[8] Liang CK, Chu CS, Chen LK (2023). Impacts of loneliness and social isolation on healthy longevity in older adults. Loneliness and Social Isolation in Old Age.

[9] Eldadah BA (2010). Fatigue and fatigability in older adults. PM R.

[10] Lisko I, Kulmala J, Annetorp M, Ngandu T, Mangialasche F, Kivipelto M (2021). How can dementia and disability be prevented in older adults: where are we today and where are we going?. J Intern Med.

[11] Arrighi HM, Gélinas Isabelle, McLaughlin TP, Buchanan J, Gauthier S (2013). Longitudinal changes in functional disability in Alzheimer's disease patients. Int Psychogeriatr.

[12] Blaum CS, Xue QL, Michelon E, Semba RD, Fried LP (2005). The association between obesity and the frailty syndrome in older women: the Women's Health and Aging Studies. J Am Geriatr Soc.

[13] Chen TY, Edwards JD, Janke MC (2015). The effects of the a matter of balance program on falls and physical risk of falls, Tampa, Florida, 2013. Prev Chronic Dis.

[14] Shubert Tiffany E, Smith Matthew Lee, Ory Marcia G, Clarke Cristine B, Bomberger Stephanie A, Roberts Ellen, Busby-Whitehead Jan (2014). Translation of the otago exercise program for adoption and implementation in the United States. Front Public Health.

[15] Ory MG, Smith ML, Jiang L, Lee R, Chen S, Wilson AD, Stevens JA, Parker EM (2014). Fall prevention in community settings: results from implementing stepping on in three States. Front Public Health.

[16] Logghe IHJ, Verhagen AP, Rademaker ACHJ, Bierma-Zeinstra SMA, van Rossum E, Faber MJ, Koes BW (2010). The effects of Tai Chi on fall prevention, fear of falling and balance in older people: a meta-analysis. Prev Med.

[17] York SC, Shumway-Cook A, Silver IF, Morrison AC (2011). A translational research evaluation of the Stay Active and Independent for Life (SAIL) community-based fall prevention exercise and education program. Health Promot Pract.

[18] Ehrenreich H, Pike M, Hohman K, Kaniewski M, Longjohn M, Myers G, Lee R (2014). CDC and YMCA: A Promising Partnership for Delivering Fall Prevention Programing. Front Public Health.

[19] Schepens SL, Panzer V, Goldberg A (2011). Randomized controlled trial comparing tailoring methods of multimedia-based fall prevention education for community-dwelling older adults. Am J Occup Ther.

[20] Szanton SL, Thorpe RJ, Boyd C, Tanner EK, Leff B, Agree E, Xue Q, Allen JK, Seplaki CL, Weiss CO, Guralnik JM, Gitlin LN (2011). Community aging in place, advancing better living for elders: a bio-behavioral-environmental intervention to improve function and health-related quality of life in disabled older adults. J Am Geriatr Soc.

[21] Panzer V, Burleson JA, Wakefield D, Into F, Wolfson L (2008). Can a multimedia fall prevention treatment program change behavior and prevent falls? Journal of the American Geriatrics Society. , p.S167. ISSN. Journal of the American Geriatrics Society.

[22] Li F, Harmer P, Voit J, Chou L (2021). Implementing an online virtual falls prevention intervention during a public health pandemic for older adults with mild cognitive impairment: a feasibility trial. Clin Interv Aging.

[23] Shubert TE, Chokshi A, Mendes VM, Grier S, Buchanan H, Basnett J, Smith ML (2020). Stand Tall-a virtual translation of the Otago exercise program. J Geriatr Phys Ther.

[24] Martin A, Candow D (2019). Effects of online yoga and tai chi on physical health outcome measures of adult informal caregivers. Int J Yoga.

[25] Wehner C, Blank C, Arvandi M, Wehner C, Schobersberger W (2021). Effect of Tai Chi on muscle strength, physical endurance, postural balance and flexibility: a systematic review and meta-analysis. BMJ Open Sport Exerc Med.

[26] Mohamed Hassan Saleh N, El-Gilany AH, Noshy Abd El-Aziz Mohamed H, Mahmoud Elsakhy N (2022). Effect of matter of balance program on improving balance and reducing fear of falls among community-dwelling older adults. Egypt J Health Care.

[27] Haynes M, League P, Neault G (2014). A matter of balance: older adults taking control of falls by building confidence. Front Public Health.

[28] Reynolds L, Buchanan BL, Alexander JL, Bordenave E (2019). Effectiveness of a matter of balance program within an assisted living community. Physical & Occupational Therapy In Geriatrics.

[29] Sartor-Glittenberg C, Bordenave E, Bay C, Bordenave L, Alexander JL (2018). Effect of a matter of balance programme on avoidance behaviour due to fear of falling in older adults. Psychogeriatrics.

[30] Wolfe ES, Arabian SS, Breeze JL, Bugaev N (2018). Evaluating the effectiveness of the translated "A Matter of Balance" fall prevention program materials for non-English-speaking participants. J Trauma Nurs.

[31] Howland J, Shankar KN, Peterson EW, Taylor AA (2015). Savings in acute care costs if all older adults treated for fall-related injuries completed matter of balance. Inj Epidemiol.

[32] Bhatt T, Wang Y, Wang S, Kannan L (2021). Perturbation training for fall-risk reduction in healthy older adults: interference and generalization to opposing novel perturbations post intervention. Front Sports Act Living.

[33] Kannan L, Bhatt T, Zhang A, Ajilore O (2022). Association of balance control mechanisms with brain structural integrity in older adults with mild cognitive impairment. Neurosci Lett.

[34] Kannan L, Bhatt T, Ajilore O (2023). Cerebello-cortical functional connectivity may regulate reactive balance control in older adults with mild cognitive impairment. Front Neurol.

[35] Kannan L, Bhatt T (2021). Associations of dual task exergaming with cognitive-motor interference in older adults with mild cognitive impairment: a single-arm pilot study. JAR Life.

[36] Moreira NB, Rodacki ALF, Costa SN, Pitta A, Bento PCB (2021). Perceptive-cognitive and physical function in prefrail older adults: exergaming versus traditional multicomponent training. Rejuvenation Res.

[37] Zhao Y, Feng H, Wu X, Du Y, Yang X, Hu M, Ning H, Liao L, Chen H, Zhao Y (2020). Effectiveness of exergaming in improving cognitive and physical function in people with mild cognitive impairment or dementia: systematic review. JMIR Serious Games.

[38] Dermody G, Whitehead L, Wilson G, Glass C (2020). The role of virtual reality in improving health outcomes for community-dwelling older adults: systematic review. J Med Internet Res.

[39] Kamnardsiri T, Phirom K, Boripuntakul S, Sungkarat S (2021). An interactive physical-cognitive game-based training system using kinect for older adults: development and usability study. JMIR Serious Games.

[40] Kwan RY, Lee D, Lee PH, Tse M, Cheung DS, Thiamwong L, Choi KS (2020). Effects of an mHealth brisk walking intervention on increasing physical activity in older people with cognitive frailty: pilot randomized controlled trial. JMIR Mhealth Uhealth.

[41] Stawarz K, Liang IJ, Alexander L, Carlin A, Wijekoon A, Western MJ (2023). Exploring the potential of technology to promote exercise snacking for older adults who are prefrail in the home setting: user-centered design study. JMIR Aging.

[42] Subramaniam S, Kitsiou S, Bhatt T (2023). Remote teleassessment and telerehabilitation of a comprehensive exercise training protocol for older adults: design and methodology of a usability protocol. Internet j allied health sci pract.

[43] Xue QL, Bandeen-Roche K, Varadhan R, Zhou J, Fried LP (2008). Initial manifestations of frailty criteria and the development of frailty phenotype in the women's health and aging study II. J Gerontol A Biol Sci Med Sci.

[44] Stenholm S, Ferrucci L, Vahtera J, Hoogendijk EO, Huisman M, Pentti J, Lindbohm JV, Bandinelli S, Guralnik JM, Kivimäki Mika (2019). Natural course of frailty components in people who develop frailty syndrome: evidence from two cohort studies. J Gerontol A Biol Sci Med Sci.

[45] de Aquino MPM, de Oliveira Cirino NT, Lima CA, de Miranda Ventura M, Hill K, Perracini MR (2022). The four square step test is a useful mobility tool for discriminating older persons with frailty syndrome. Exp Gerontol.

[46] Filiatrault J, Gauvin L, Fournier M, Parisien M, Robitaille Y, Laforest S, Corriveau H, Richard L (2007). Evidence of the psychometric qualities of a simplified version of the activities-specific balance confidence scale for community-dwelling seniors. Arch Phys Med Rehabil.

[47] Yang C, Mo Y, Cao X, Zhu S, Wang X, Wang X (2023). Reliability and validity of the tinetti performance oriented mobility assessment in Chinese community-dwelling older adults. Geriatr Nurs.

[48] Tinetti ME (1986). Performance-oriented assessment of mobility problems in elderly patients. J Am Geriatr Soc.

[49] Millor N, Lecumberri P, Gómez M, Martínez-Ramírez A, Izquierdo M (2013). An evaluation of the 30-s chair stand test in older adults: frailty detection based on kinematic parameters from a single inertial unit. J Neuroeng Rehabil.

[50] Bohannon RW, Crouch RH (2019). Two-minute step test of exercise capacity: systematic review of procedures, performance, and clinimetric properties. J Geriatr Phys Ther.

[51] Yardley L, Redfern MS (2001). Psychological factors influencing recovery from balance disorders. J Anxiety Disord.

[52] Buccello-Stout RR, Bloomberg JJ, Cohen HS, Whorton EB, Weaver GD, Cromwell RL (2008). Effects of sensorimotor adaptation training on functional mobility in older adults. J Gerontol B Psychol Sci Soc Sci.

