# Supplementary material for: Gaming-Based Tele-Exercise Program to Improve Physical Function in Frail Older Adults: Feasibility Randomized Controlled Trial
Source: J Med Internet Res. 2024 Nov 27;26:e56810. doi: 10.2196/56810 (PMC11635319; doi:10.2196/56810)
Supplement: Multimedia Appendix 1 [file jmir_v26i1e56810_app1.pdf]

| CONSORT-EHEALTH Checklist V1.6.2 Report | Manuscript Number | 56810 |
|---|---|---|
| **(based on CONSORT-EHEALTH V1.6), available at [http://tinyurl.com/consort-ehealth-v1-6].** | | |
| | | |
| **Date completed** | | |
| 10/30/2024 4:50:39 | | |
| **by** | | |
| Lakshmi | | |
| | | |
| Gaming-Based Tele-Exercise Program to Improve Physical Function in Frail Older Adults: Feasibility Randomized Controlled Trial | | |
| TITLE | | |
| **1a-i) Identify the mode of delivery in the title** | | |
| "Gaming-Based Tele-Exercise" | | |
| **1a-ii) Non-web-based components or important co-interventions in title** | | |
| "Gaming-Based Tele-Exercise" | | |
| **1a-iii) Primary condition or target group in the title** | | |
| The study included "Frail Older Adults" | | |
| ABSTRACT | | |
| **1b-i) Key features/functionalities/components of the intervention and comparator in the METHODS section of the ABSTRACT** | | |
| "CogXergaming" "Matter of Balance" | | |
| **1b-ii) Level of human involvement in the METHODS section of the ABSTRACT** | | |
| Intervention is researcher administered and is explained in detail under the methods section | | |
| **1b-iii) Open vs. closed, web-based (self-assessment) vs. face-to-face assessments in the METHODS section of the ABSTRACT** | | |
| "supervised tele-exercises" | | |
| **1b-iv) RESULTS section in abstract must contain use data** | | |
| "dropped out of the study" | | |
| **1b-v) CONCLUSIONS/DISCUSSION in abstract for negative trials** | | |
| "CogXergaming is feasible and applicable" | | |
| INTRODUCTION | | |
| **2a-i) Problem and the type of system/solution** | | |
| "this pilot used a NIH (National Institutes of Health) stage 1 model, randomized control trial to investigate the feasibility and effectiveness of a noninteractive, virtually delivered (through Zoom) exergame (ie, CogXergaming) compared with virtually delivered (also through Zoom) MOB on physical function (ie, balance control assessed through Four Square Step Test [FSST], self-efficacy through Activities-Specific Balance Confidence [ABC] scale, gait function through Tinetti Performance Oriented Mobility Assessment [POMA], muscle strength through 30-second chair stand test [30-CST], and endurance through 2-minute step test) among pre-frail older adults" | | |
| **2a-ii) Scientific background, rationale: What is known about the (type of) system** | | |
| "Our previous work administered a tele-based assessment and training method called CogXergaming, that is safe to deliver to older adults [42]. The training involves remotely monitoring older adults who mimic a combination of exergame videos (noninteractive) that targets training of balance control, endurance, cognitive-motor function, and mobility [42]. The observed benefits suggest initial safety and feasibility implications in older adults . Adopting a prudent, stepwise approach to assessing the feasibility of a similar training paradigm in pre-frail older adults is essential for minimizing potential harm to frail individuals. This approach will ensure rigorous evaluation to establish the safety and effectiveness of interventions before broader implementation in higher-risk populations." | | |
| **Does your paper address CONSORT subitem 2b?** | | |
| "We hypothesized that this pilot study will be feasible and that participants will demonstrate adherence to the CogXergaming program. In addition, the study will be explore between-group (CogXergaming vs MOB) differences to gather preliminary data on the comparative effectiveness of the 2 interventions." | | |
| METHODS | | |
| **3a) CONSORT: Description of trial design (such as parallel, factorial) including allocation ratio** | | |
| "Pre-frail older adult were recruited. They were characterized as pre-frail if at least 2 out of the 3 criteria were present: (1) self-reported unintentional weight loss (>10 lbs) in the last 1 year [1], (2) self-reported general fatigue >3 on rate of perceived exertion (ie, according to Borg's scale ranging from 1-10 with 10 being the maximum perceived exertion) at rest [1], and (3) reduced physical activity levels (ie, exercise less than 3 hours a week) [1]. These criteria are recognized as preliminary indicators of pre-frailty [43,44] and were selected as feasible measures for remote screening, aligning with the study's practical considerations and the goal of targeting pre-frail individuals for our intervention." | | |
| **3b) CONSORT: Important changes to methods after trial commencement (such as eligibility criteria), with reasons** | | |
| "Participants were required to possess a laptop or tablet or personal computer with stable internet provision. This decision was made to focus on the need for larger screens that could effectively support the tasks and activities involved in our study. In addition, participants must be able to walk more than 30 feet, stand without an assistive device for at least 5 minutes (length of an exergame was approximately 1-3 minutes), and understand English. Participants were excluded if they self-reported any acute and uncontrolled chronic medical illnesses such as cardiolovascular or pulmonary conditions; were recently hospitalized (<3 months); or had a diagnosed neurological condition such as a stroke or Parkinson's disease. They were also excluded if their weight exceeded 100 kgs and if they were using sedatives or undergoing psychiatric treatment that could affect their cognitive and physical performance during training sessions." | | |
| **3b-i) Bug fixes, Downtimes, Content Changes** | | |
| No unexpected events occurred. Therefore, we have nothing to report. "Safety was a priority; therefore, the researcher inspected the environment through the Zoom call and asked the participant to clear away any objects that could potentially cause injury. The researcher instructed the participant to choose a room with good lighting. Besides the break that automatically occurred between subsessions, additional rest breaks were provided upon participant requested ones." | | |
| **4a) CONSORT: Eligibility criteria for participants** | | |
| "Pre-frail older adult were recruited. They were characterized as pre-frail if at least 2 out of the 3 criteria were present: (1) self-reported unintentional weight loss (>10 lbs) in the last 1 year [1], (2) self-reported general fatigue >3 on rate of perceived exertion (ie, according to Borg's scale ranging from 1-10 with 10 being the maximum perceived exertion) at rest [1], and (3) reduced physical activity levels (ie, exercise less than 3 hours a week) [1]. These criteria are recognized as preliminary indicators of pre-frailty [43,44] and were selected as feasible measures for remote screening, aligning with the study's practical considerations and the goal of targeting pre-frail individuals for our intervention. Participants were required to possess a laptop or tablet or personal computer with stable internet provision. This decision was made to focus on the need for larger screens that could effectively support the tasks and activities involved in our study. In addition, participants must be able to walk more than 30 feet, stand without an assistive device for at least 5 minutes (length of an exergame was approximately 1-3 minutes), and understand English. Participants were excluded if they self-reported any acute and uncontrolled chronic medical illnesses such as cardiolovascular or pulmonary conditions; were recently hospitalized (<3 months); or had a diagnosed neurological condition such as a stroke or Parkinson's disease. They were also excluded if their weight exceeded 100 kgs and if they were using sedatives or undergoing psychiatric treatment that could affect their cognitive and physical performance during training sessions." | | |
| **4a-i) Computer / Internet literacy** | | |
| "All sessions were conducted over Health Insurance Portability and Accountability Act (HIPAA) compliant Zoom." | | |
| **4a-ii) Open vs. closed, web-based vs. face-to-face assessments:** | | |
| "Participants were recruited from rehabilitation clinics and senior housing sites in the Chicago metropolitan area." | | |
| **4a-iii) Information giving during recruitment** | | |
| "This NIH stage 1, pilot study involved 50 community-dwelling older adults over the age of 60 interested in the study with 45 older adult enrolled after virtually obtaining a written informed consent (through Docusign). The Institutional Review Board (IRB) of the University of Illinois at Chicago approved the study ( #2020-0280). The study was also registered on clinicaltrials.gov (#NCT04534686)." | | |
| **4b) CONSORT: Settings and locations where the data were collected** | | |

| | | |
|---|---|---|
| For CogXergaming "Safety was a priority; therefore, the researcher inspected the environment through the Zoom call and asked the participant to clear away any objects that could potentially cause injury. The researcher instructed the participant to choose a room with good lighting. Besides the break that automatically occurred between subsessions, additional rest breaks were provided upon participant requested ones."<br><br>For Matter of Balance "All sessions were conducted over Health Insurance Portability and Accountability Act (HIPAA) compliant Zoom. Participants were trained in groups of 10. Each session lasted 120 minutes and was performed 1 time per week for 8 weeks. The researcher administering MOB was certified to perform MOB training. Each MOB session included a discussion of practical coping strategies, group problem-solving, home safety evaluation strength exercises of the lower limb, coordination exercises, and standing balance exercises [26]." | | |
| **4b-i) Report if outcomes were (self-)assessed through online questionnaires** | | |
| "All tele-based assessment tests have shown to be feasible and were described in our previous study [42]. For the current study, we used time taken to complete the FSST with lower numbers (in seconds), indicating better performance to assess balance control [45]. Subjective self-efficacy questionnaire was assessed by the ABC scale with higher values indicating higher confidence levels [46]. Gait function was assessed using the Tinetti POMA with higher scores indicating better performance [47,48]. To assess muscle strength, we used the number sit-to-stands on the 30-CST with higher values indicating greater lower-limb muscle strength [49]. Endurance was assessed using the total number of steps completed on the 2-minute step test with higher numbers indicating better endurance [50]." | | |
| **4b-ii) Report how institutional affiliations are displayed** | | |
| Participants were reimbursed with amazon gift cards and their participation was voluntary. They could drop out of the study at any point. | | |
| **5) CONSORT: Describe the interventions for each group with sufficient details to allow replication, including how and when they were actually administered** | | |
| **5-i) Mention names, credential, affiliations of the developers, sponsors, and owners** | | |
| "We thank the members of the Cognitive Motor Balance Rehabilitation laboratory for assisting with study recruitment and procedure administration. This work was supported by the National Institute on Aging of the National Institutes of Health (NIH; grant number R24AG064191). The funder had no role in the design and conduct of the study; collection, management, analysis, and interpretation of the data; preparation, review, or approval of the manuscript; or decision to submit the manuscript for publication." | | |
| **5-ii) Describe the history/development process** | | |
| "Our previous work administered a tele-based assessment and training method called CogXergaming, that is safe to deliver to older adults [42]. " | | |
| **5-iii) Revisions and updating** | | |
| "More details of this method have been previously published and have shown feasibility [42]." | | |
| **5-iv) Quality assurance methods** | | |
| "Participants possessed either a tablet or laptop to participate in the intervention activities. However, the specific data on the distribution between tablet or laptop usage among participants was not obtained. While acknowledging the importance of reporting device distribution for comprehensive study outcomes, our focus was primarily on the suitability of tablet or laptop devices to achieve the study objectives effectively.<br>Of the 45 participants enrolled in the study, 4 participants from CogXergaming group and 5 participants from MOB group lost contact after signing the consent form and did not receive their respective intervention (Figure 1). This primarily was a recruitment failure. Both the tele-based assessment and training were performed through the HIPAA–compliant Zoom application. An individualized Zoom link was sent to each CogXergaming participant for their assessments and training, and a common Zoom link was sent individually for MOB group sessions to protect participants' personal information. All participants were provided with detailed instructions over the phone, guiding them through the process of connecting to the Zoom platform. This approach facilitated seamless participation in virtual sessions, with no notable issues encountered during setup. All assessment and the training sessions were recorded for post-session analyses." | | |
| **5-v) Ensure replicability by publishing the source code, and/or providing screenshots/screen-capture video, and/or providing flowcharts of the algorithms used** | | |
| "More details of this method have been previously published and have shown feasibility [42]." | | |
| **5-vi) Digital preservation** | | |
| ""More details of this method have been previously published and have shown feasibility [42]."" | | |
| **5-vii) Access** | | |
| Participants were reimbursed with amazon gift cards and their participation was voluntary. They could drop out of the study at any point.<br>"The researcher shared their screen, and participants were required to have their video turned on to enable the researcher to observe their performance." | | |
| **5-viii) Mode of delivery, features/functionalities/components of the intervention and comparator, and the theoretical framework** | | |
| For CogXergaming "Participants were trained one-on-one for 90 minutes per day and 3 times per week for 6 weeks (for a total of 18 sessions)." "The researcher shared their screen, and participants were required to have their video turned on to enable the researcher to observe their performance."<br>For Matter of Balance "Participants were trained in groups of 10. Each session lasted 120 minutes and was performed 1 time per week for 8 weeks. The researcher administering MOB was certified to perform MOB training. " | | |
| **5-ix) Describe use parameters** | | |
| For CogXergaming "Safety was a priority; therefore, the researcher inspected the environment through the Zoom call and asked the participant to clear away any objects that could potentially cause injury. The researcher instructed the participant to choose a room with good lighting. Besides the break that automatically occurred between subsessions, additional rest breaks were provided upon participant requested ones." | | |
| **5-x) Clarify the level of human involvement** | | |
| "The researcher shared their screen, and participants were required to have their video turned on to enable the researcher to observe their performance." | | |
| **5-xi) Report any prompts/reminders used** | | |
| "These research assistants did not interact with participants assigned to the other intervention to prevent any potential bias in intervention delivery. However, we also had attrition (n=9) between consenting and start of intervention due to losing contact with participants (unreachable, phone number changed, changed mind, and not interested)." | | |
| **5-xii) Describe any co-interventions (incl. training/support)** | | |
| There were no co-interventions or support provided in addition to CogXergaming or Matter of Balance | | |
| **6a) CONSORT: Completely defined pre-specified primary and secondary outcome measures, including how and when they were assessed** | | |
| "All tele-based assessment tests have shown to be feasible and were described in our previous study [42]. For the current study, we used time taken to complete the FSST with lower numbers (in seconds), indicating better performance to assess balance control [45]. Subjective self-efficacy questionnaire was assessed by the ABC scale with higher values indicating higher confidence levels [46]. Gait function was assessed using the Tinetti POMA with higher scores indicating better performance [47,48]. To assess muscle strength, we used the number sit-to-stands on the 30-CST with higher values indicating greater lower-limb muscle strength [49]. Endurance was assessed using the total number of steps completed on the 2-minute step test with higher numbers indicating better endurance [50]." | | |
| **6a-i) Online questionnaires: describe if they were validated for online use and apply CHERRIES items to describe how the questionnaires were designed/deployed** | | |
| "More details of this method have been previously published and have shown feasibility [42]." | | |
| **6a-ii) Describe whether and how "use" (including intensity of use/dosage) was defined/measured/monitored** | | |
| "Participants possessed either a tablet or laptop to participate in the intervention activities. However, the specific data on the distribution between tablet or laptop usage among participants was not obtained. While acknowledging the importance of reporting device distribution for comprehensive study outcomes, our focus was primarily on the suitability of tablet or laptop devices to achieve the study objectives effectively.<br>Of the 45 participants enrolled in the study, 4 participants from CogXergaming group and 5 participants from MOB group lost contact after signing the consent form and did not receive their respective intervention (Figure 1). This primarily was a recruitment failure. Both the tele-based assessment and training were performed through the HIPAA–compliant Zoom application. An individualized Zoom link was sent to each CogXergaming participant for their assessments and training, and a common Zoom link was sent individually for MOB group sessions to protect participants' personal information. All participants were provided with detailed instructions over the phone, guiding them through the process of connecting to the Zoom platform. This approach facilitated seamless participation in virtual sessions, with no notable issues encountered during setup. All assessment and the training sessions were recorded for post-session analyses." | | |
| **6a-iii) Describe whether, how, and when qualitative feedback from participants was obtained** | | |
| For Matter of Balance "Participants were trained in groups of 10. Each session lasted 120 minutes and was performed 1 time per week for 8 weeks." | | |
| **6b) CONSORT: Any changes to trial outcomes after the trial commenced, with reasons** | | |

| | | |
|---|---|---|
| For CogXergaming "Safety was a priority; therefore, the researcher inspected the environment through the Zoom call and asked the participant to clear away any objects that could potentially cause injury. The researcher instructed the participant to choose a room with good lighting. Besides the break that automatically occurred between subsessions, additional rest breaks were provided upon participant requested ones."<br><br>For Matter of Balance "All sessions were conducted over Health Insurance Portability and Accountability Act (HIPAA) compliant Zoom. Participants were trained in groups of 10. Each session lasted 120 minutes and was performed 1 time per week for 8 weeks. The researcher administering MOB was certified to perform MOB training. Each MOB session included a discussion of practical coping strategies, group problem-solving, home safety evaluation strength exercises of the lower limb, coordination exercises, and standing balance exercises [26]." | | |
| **7a) CONSORT: How sample size was determined** | | |
| **7a-i) Describe whether and how expected attrition was taken into account when calculating the sample size** | | |
| "The study was not powered for formal efficacy analyses due to the limited sample size and exploratory nature of the investigation" | | |
| **7b) CONSORT: When applicable, explanation of any interim analyses and stopping guidelines** | | |
| "All tele-based assessment tests have shown to be feasible and were described in our previous study [42]. For the current study, we used time taken to complete the FSST with lower numbers (in seconds), indicating better performance to assess balance control [45]. Subjective self-efficacy questionnaire was assessed by the ABC scale with higher values indicating higher confidence levels [46]. Gait function was assessed using the Tinetti POMA with higher scores indicating better performance [47,48]. To assess muscle strength, we used the number sit-to-stands on the 30-CST with higher values indicating greater lower-limb muscle strength [49]. Endurance was assessed using the total number of steps completed on the 2-minute step test with higher numbers indicating better endurance [50]." | | |
| **8a) CONSORT: Method used to generate the random allocation sequence** | | |
| "Out of 50 OA who met the inclusion criteria, 45were included in the study and signed the consent form. Participants were randomized to either of the training groups (n=22 in CogXergaming and n=23 in MOB) by flipping a coin by a research coordinator. This is a simple and transparent randomization technique based on the outcome of a coin toss. A specific research assistant was assigned to administer the CogXergaming intervention, while a different research assistant, trained in delivering the MOB training, administered the MOB intervention. These research assistants did not interact with participants assigned to the other intervention to prevent any potential bias in intervention delivery. However, we also had attrition (n=9) between consenting and start of intervention due to losing contact with participants (unreachable, phone number changed, changed mind, and not interested)." | | |
| **8b) CONSORT: Type of randomisation; details of any restriction (such as blocking and block size)** | | |
| "Participants were randomized to either of the training groups (n=22 in CogXergaming and n=23 in MOB) by flipping a coin by a research coordinator." | | |
| **9) CONSORT: Mechanism used to implement the random allocation sequence (such as sequentially numbered containers), describing any steps taken to conceal the sequence until interventions were assigned** | | |
| "Participants were randomized to either of the training groups (n=22 in CogXergaming and n=23 in MOB) by flipping a coin by a research coordinator." | | |
| **10) CONSORT: Who generated the random allocation sequence, who enrolled participants, and who assigned participants to interventions** | | |
| "Participants were randomized to either of the training groups (n=22 in CogXergaming and n=23 in MOB) by flipping a coin by a research coordinator." | | |
| **11a) CONSORT: Blinding - If done, who was blinded after assignment to interventions (for example, participants, care providers, those assessing outcomes) and how** | | |
| **11a-i) Specify who was blinded, and who wasn't** | | |
| "These research assistants did not interact with participants assigned to the other intervention to prevent any potential bias in intervention delivery." | | |
| **11a-ii) Discuss e.g., whether participants knew which intervention was the "intervention of interest" and which one was the "comparator"** | | |
| "This is a simple and transparent randomization technique based on the outcome of a coin toss. A specific research assistant was assigned to administer the CogXergaming intervention, while a different research assistant, trained in delivering the MOB training, administered the MOB intervention. These research assistants did not interact with participants assigned to the other intervention to prevent any potential bias in intervention delivery." | | |
| **11b) CONSORT: If relevant, description of the similarity of interventions** | | |
| There was no similarity between the interventions | | |
| **12a) CONSORT: Statistical methods used to compare groups for primary and secondary outcomes** | | |
| "All analysis was performed using SPSS (version 24, IBM Corp). To assess demographic differences, an independent t test with Bonferroni correction for all characteristics except gender. A chi-square test of independence for gender was conducted. It should be noted that this pilot trial was designed to primarily assess the feasibility and safety of a tele-exercise intervention (CogXergaming) in pre-frail older adults. The study was not powered for formal efficacy analyses due to the limited sample size and exploratory nature of the investigation. A 2 □ 2 repeated-measure ANOVA was performed to determine the time effect (pre- and posttraining), group effect (CogXergaming vs MOB), and the time □ group interaction on all teleassessments (ie, balance control and confidence, gait function, muscle strength, endurance). Finally, median duration of training sessions for the CogXergaming group was computed to address feasibility of training. Significant main effects were resolved by post hoc tests with Bonferroni correction with significance level □=.05." | | |
| **12a-i) Imputation techniques to deal with attrition / missing values** | | |
| "Of the 18 people who were randomized to the CogXergaming group, 15, that is, 83% successfully completed the intervention. All the attrition from participation in the treatments tested occurred within the first week of both programs. During the first week of CogXergaming, 3 participants (16 %) withdrew or dropped out due to lack of time and commitment (eg, got a new job, caregiving duties). Similarly, of the 18 people randomized to MOB, 16 (88%) completed the program. Specifically, 2 people (11%) were not interested in continuing after attending the first session of the MOB group. We did not experience attrition from treatment in either group after participants completed the first week of training." | | |
| **12b) CONSORT: Methods for additional analyses, such as subgroup analyses and adjusted analyses** | | |
| Not applicable because we have used only those participants who completed all tests in the study. | | |
| RESULTS | | |
| **13a) CONSORT: For each group, the numbers of participants who were randomly assigned, received intended treatment, and were analysed for the primary outcome** | | |
| "Of the 45 participants enrolled in the study, 4 participants from CogXergaming group and 5 participants from MOB group lost contact after signing the consent form and did not receive their respective intervention (Figure 1). "<br>"Of the 18 people who were randomized to the CogXergaming group, 15, that is, 83% successfully completed the intervention. All the attrition from participation in the treatments tested occurred within the first week of both programs. During the first week of CogXergaming, 3 participants (16 %) withdrew or dropped out due to lack of time and commitment (eg, got a new job, caregiving duties). Similarly, of the 18 people randomized to MOB, 16 (88%) completed the program. Specifically, 2 people (11%) were not interested in continuing after attending the first session of the MOB group. We did not experience attrition from treatment in either group after participants completed the first week of training." | | |
| **13b) CONSORT: For each group, losses and exclusions after randomisation, together with reasons** | | |
| "Of the 45 participants enrolled in the study, 4 participants from CogXergaming group and 5 participants from MOB group lost contact after signing the consent form and did not receive their respective intervention (Figure 1). " | | |
| **13b-i) Attrition diagram** | | |
| Figure 1. Research design. shows the number of participants involved in the study. | | |
| **14a) CONSORT: Dates defining the periods of recruitment and follow-up** | | |
| Figure 1. Research design. shows the number of participants involved in the study. | | |
| **14a-i) Indicate if critical "secular events" fell into the study period** | | |
| This is not applicable because there were no secular events or any conditions mentioned above. | | |
| **14b) CONSORT: Why the trial ended or was stopped (early)** | | |
| The trial did not end or stopped early, therefore, this part is not applicable. | | |
| **15) CONSORT: A table showing baseline demographic and clinical characteristics for each group** | | |
| Table 1: Participant demographic characteristics for both groups. | | |
| **15-i) Report demographics associated with digital divide issues** | | |
| Table 1: Participant demographic characteristics for both groups. | | |
| **16a) CONSORT: For each group, number of participants (denominator) included in each analysis and whether the analysis was by original assigned groups** | | |
| **16-i) Report multiple "denominators" and provide definitions** | | |
| Participant descriptive characteristics are shown in Table 1. No significant group differences in participant characteristics were observed (P>.05). | | |
| **16-ii) Primary analysis should be intent-to-treat** | | |
| Not relevant to the study. | | |
| **17a) CONSORT: For each primary and secondary outcome, results for each group, and the estimated effect size and its precision (such as 95% confidence interval)** | | |

| | | |
|---|---|---|
| "The study was not powered for formal efficacy analyses due to the limited sample size and exploratory nature of the investigation. " | | |
| **17a-i) Presentation of process outcomes such as metrics of use and intensity of use** | | |
| This is not relevant as the sessions were not timed and enough break were given to participants. | | |
| **17b) CONSORT: For binary outcomes, presentation of both absolute and relative effect sizes is recommended** | | |
| We did not have any binary outcomes included in the study. | | |
| **18) CONSORT: Results of any other analyses performed, including subgroup analyses and adjusted analyses, distinguishing pre-specified from exploratory** | | |
| This was a randomized control trial with a specific hypothesis driven. | | |
| **18-i) Subgroup analysis of comparing only users** | | |
| "These research assistants did not interact with participants assigned to the other intervention to prevent any potential bias in intervention delivery. " | | |
| **19) CONSORT: All important harms or unintended effects in each group** | | |
| There were no harms or unintended consequences observed during the course of study. | | |
| **19-i) Include privacy breaches, technical problems** | | |
| We followed specific data safety and monitoring to keep the data secure and private. No breech of security was observed. | | |
| **19-ii) Include qualitative feedback from participants or observations from staff/researchers** | | |
| All sessions were recorded and no specific qualitative analysis was performed or observed that was required to be reported . | | |
| DISCUSSION | | |
| **20) CONSORT: Trial limitations, addressing sources of potential bias, imprecision, multiplicity of analyses** | | |
| **20-i) Typical limitations in ehealth trials** | | |
| "Apart from the benefits observed, acknowledging the limitations of the study is essential. First, the relatively low sample size in our pilot study meant that the study did not have the statistical power to enable valid between group efficacy testing. The differential within-group improvements on 3 of the outcomes is promising and warrants follow-up in a fully powered future. The nonequivalence between the groups in our study may have influenced the outcomes observed. However, since this was a pilot study aimed at evaluating the feasibility of a noninteractive exergaming training, future research efforts may benefit from designing equivalent-based training programs to determine the efficacy of the intervention more accurately. Second, caution must be used in generalizing the observed results to other populations. Higher sample size in future studies may provide more robust and representative data, particularly in terms of the scope and methods used to identify pre-frail individuals. Third, the intervention implicitly challenged cognitive function but did not include comprehensive examination of cognitive aspects associated with increased risk of falling (like reaction time, working memory, executive function). Further exploration of these additional outcomes is therefore warranted. Finally, although the study included pre-frail older adults , we did not differentiate between physical frailty, cognitive frailty, or both. Future investigations may benefit from classifying participants based on these distinct frailty profiles to assess intervention effects more powerfully." | | |
| **21) CONSORT: Generalisability (external validity, applicability) of the trial findings** | | |
| **21-i) Generalizability to other populations** | | |
| "In conclusion, while our NIH stage 1 pilot study provides valuable insights into the feasibility of a noninteractive exergaming intervention, future research efforts should address these limitations to advance our understanding of the intervention's efficacy and generalizability." | | |
| **21-ii) Discuss if there were elements in the RCT that would be different in a routine application setting** | | |
| There were no no elements in the RCT that was different in a routine application setting. | | |
| **22) CONSORT: Interpretation consistent with results, balancing benefits and harms, and considering other relevant evidence** | | |
| **22-i) Restate study questions and summarize the answers suggested by the data, starting with primary outcomes and process outcomes (use)** | | |
| "This pilot randomized controlled trial investigated the feasibility and effectiveness of a tele-based, noninteractive CogXergaming training compared with tele-based MOB program on physical function in pre-frail older adults . The results showed that the intervention was feasible as shown by tolerability of the intervention with a lack of any expected and unexpected intervention-induced adverse events. The intervention also showed promise of being effective as seen by the significantly and differentially greater improvements in physical function within the CogXergaming group. Specifically, we observed significant and differential within group improvements within the CogXergaming group in balance control and confidence, gait function, and lower limb muscle strength. While both groups showed significant improvement in endurance, the benefits were greater among the MOB group. These results suggest that the tele-based, noninteractive CogXergaming intervention has the potential to improve physical function among pre-frail older adults ." | | |
| **22-ii) Highlight unanswered new questions, suggest future research** | | |
| "In conclusion, while our NIH stage 1 pilot study provides valuable insights into the feasibility of a noninteractive exergaming intervention, future research efforts should address these limitations to advance our understanding of the intervention's efficacy and generalizability." | | |
| Other information | | |
| **23) CONSORT:  Registration number and name of trial registry** | | |
| "The study was also registered on clinicaltrials.gov (#NCT04534686). " | | |
| **24) CONSORT: Where the full trial protocol can be accessed, if available** | | |
| "The study was also registered on clinicaltrials.gov (#NCT04534686)." | | |
| **25) CONSORT: Sources of funding and other support (such as supply of drugs), role of funders** | | |
| "NIH; grant number R24AG064191" | | |
| **X26-i) Comment on ethics committee approval** | | |
| "The Institutional Review Board (IRB) of the University of Illinois at Chicago approved the study ( #2020-0280). The study was also registered on clinicaltrials.gov (#NCT04534686). " | | |
| **x26-ii) Outline informed consent procedures** | | |
| "This NIH stage 1, pilot study involved 50 community-dwelling older adults over the age of 60 interested in the study with 45 older adult enrolled after virtually obtaining a written informed consent (through Docusign). " | | |
| **X26-iii) Safety and security procedures** | | |
| "Both the tele-based assessment and training were performed through the HIPAA–compliant Zoom application. An individualized Zoom link was sent to each CogXergaming participant for their assessments and training, and a common Zoom link was sent individually for MOB group sessions to protect participants' personal information. All participants were provided with detailed instructions over the phone, guiding them through the process of connecting to the Zoom platform. " | | |
| **X27-i) State the relation of the study team towards the system being evaluated** | | |
| We have nothing to declare. | | |
